# A survey of Post-Traumatic Stress Disorder, Anxiety and Depression among Flood Affected Populations in Kerala, India

**DOI:** 10.3126/nje.v12i2.46334

**Published:** 2022-06-30

**Authors:** Mohammad Asim, Brijesh Sathian, Edwin van Teijlingen, Ahammed A Mekkodathil, M G Ramesh Babu, Elayedath Rajesh, Rajeev Kumar N, Padam Simkhada, Indrajit Banerjee

**Affiliations:** 1 Clinical Research, Trauma and Vascular Surgery, Surgery Department, Hamad General Hospital, Doha, Qatar; 2 Geriatrics and long term care department, Rumailah Hospital, Hamad Medical Corporation, Doha, Qatar; 3 Centre for Midwifery, Maternal and Perinatal Health, Bournemouth University, Bournemouth, UK; 4 Division of Physiology, Department of Basic Medical Sciences, Manipal Academy of Higher Education [MAHE], Manipal, India; 5 Schoool of Behavioural Sciences, Mahatma Gandhi University, Kerala, India; 6 School of Human and Health Sciences, University of Huddersfield, Huddersfield HD1 3DH, UK; 7 Department of Pharmacology, SSR Medical College, Belle Rive, Mautitius

**Keywords:** Flood, Post-Traumatic Stress Disorder, Anxiety, Depression

## Abstract

**Background:**

Globally, post traumatic stress disorder (PTSD) is one of the most common psychiatric illnesses following a disaster. We aimed to evaluate the relationship between the socio-economic and flood exposure factors with PTSD, depression and anxiety among the flood-affected populations in Kerala, India.

**Methods:**

A cross-sectional household survey was conducted from November 2019 to January 2020 in Kozhikode district of Kerala, India. Adults (≥ 18 years), who were permanent residents and had been directly exposed to the flood, were invited to take part in the study. Individuals with a history of mental health issues and those who had other stressful situations in the past were excluded. The survey questionnaire was based on three screening tools: (1) PTSD Checklist for DSM-5 (PCL-5); (2) patient health questionnaire (PHQ-9); and (3) generalized anxiety disorder (GAD-7). Data included sociodemographic factors and flood exposure variables. The primary outcome variable was psychiatric morbidity (PTSD, anxiety and depression).

**Results:**

A total of 276 respondents (150 males/126 females) participated in the study. A significant correlation was observed between total score on PCL-5 and GAD-7 (r=0.339, p=0.001) and PHQ-9 (r=0.262, p=0.001). Females had significantly higher total PTSD symptom severity scores (8.24±5.88 vs. 6.07±5.22; p=0.001), severity of symptoms of intrusion (4.66±3.60 vs. 3.69±3.20; p=0.04), increased level of anxiety (2.54±1.94 vs. 1.79±1.53; p=0.001) and depression (3.02±2.26 vs. 2.04±1.67; p=0.001) compared to males. However, the gender difference for PTSD symptoms disappeared when controlling for age.

**Conclusion:**

The findings of this survey revealed that the vast majority of respondents (92 percent females and 87 percent males) still had subclinical psychiatric symptoms one year after the flood. Therefore, tailored psychological interventions are warranted to counter the long-lasting impact of flooding on the mental health of individuals.

## Introduction

Psychological disorders account for a significant amount of the world's disease burden, and are the primary cause of years spent living with a disability (Disability-Adjusted Life Years) [1]. This causes decreased productivity, and has a detrimental effect on the quality of life of the affected individuals and society as a whole [2]. Flooding has a variety of negative impacts on human health, including an increased risk of drowning, injuries, loss of property and long-lasting psychological illnesses [3]. Post-traumatic stress disorder (PTSD), depression, anxiety, and suicidal ideation are psychiatric morbidities that are often associated with flood exposure [4,5]. Notably, PTSD is the psychological illness that victims of flooding are most likely to develop [5]. Besides, sleep deprivation, emotional numbness, avoidance, and excessive arousal are the most common symptoms of PTSD which may develop and persist for longer duration following exposure to a disaster [6].

Floods are known to have a profound mental health impact, especially in poor people in low-income countries [7]. Most studies addressing the effects of flooding on mental disorders are from high-income countries, [8] whilst developing regions such as the Indian subcontinent, are less well researched. Ethnicity, frequency and extent of flooding, and social support are the key factors influencing the severity of mental illness in India [9]. Individuals who have experienced severe flooding or sudden natural disasters are most frequently diagnosed with PTSD, depression, and anxiety [10]. PTSD was reported in 30.6% children/adolescents after a natural disaster in Orissa, India [11]. According to an earlier cross-sectional study from Kashmir, having a strong support from family and friends minimizes the risk of developing symptoms of PTSD and depression after flood exposure [12]. A study conducted by Telles et al., among survivors of flood from Bihar, India demonstrated that elderly individuals are more susceptible to PTSD and depression after a natural disaster [13].

The 2015 floods in India affected 13.71 million people and inflicted enormous economic damages [14]. The Kerala flood in the year 2018 accounted for 453 deaths and around 140 people missing. The disaster displaced around 14.5 lakh people and requiring over 3000 relief camps [14]. Over one-sixth of the population of Kerala was directly affected by the flood and its aftermath. Notably, such natural disaster may have huge impact on the mental wellbeing of the victims and therefore, exploration of psychological effects on survivors is important to develop coping strategies. Though few studies have reported the prevalence of PTSD among adolescents [15] and adults [16] of 2018 flood victims in Kerala, there are no reports available on other associated psychological disorders such as depression and anxiety. Therefore, we assessed the relationship between the socio-economic and flood exposure factors with PTSD, depression and anxiety among the flood survivors in the Kozhikode district of Kerala, India.

## Methodology

### Study design and participants

A cross-sectional household survey was conducted from November 2019 to January 2020 in Kozhikode district of Kerala, India. Adults (≥ 18 years), who were permanent residents and had been directly exposed to the flood were invited to participate in the study. Individuals with a history of mental health issues and those who had other serious stressful situations in the past were excluded. A random sampling technique was chosen to select the most flood-affected wards in Kozhikode districts which was under the Kozhikode city corporation limit.

### Sample size calculation

An earlier cross-sectional study reported the prevalence of PTSD to be 22% among 100 households affected by flooding in Kerala [16]. Considering a 22% prevalence of PTSD, we would need a total of 264 participants to estimate the predicted proportion with a 5% absolute precision and a 95% confidence level in our study.We were able to get an adequate sample size consisting of of 276 respondents.

### Data collection

A single team of nine members, with a team leader and four male and four female enumerators (health workers), collected the data. A pretested semi-structured questionnaire was used to collect the socio-demographic and flood exposure factors, and the screening for psychiatric illness was done by using PTSD Checklist for DSM-5 (PCL-5); a patient health questionnaire (PHQ-9); and generalized anxiety disorder (GAD-7).

The PTSD checklist for DSM-5 (PCL-5) questionnaire contains 20 items on a Likert-type scale with a five-point range (0–4) that evaluate the symptoms of PTSD. Its total score lies between 0 to 80 and the respondent has met the diagnosis for PTSD if their score is 33 or above [17]. The presence of PTSD-related symptoms that are elevated but do not fully satisfy the diagnostic criteria for PTSD (score<33) is known as subclinical PTSD. The patient health questionnaire (PHQ-9) was used to assess depression in order to identify both current and former depression, which also correlates to the diagnosis of major depressive disorder as defined by the DSM-IV [18]. A score of 1-4 indicated that the respondents had minimal depression, a score of 5–9 indicated mild depression, a score of 10–14 indicated moderate depression, a score of 15–19 indicated fairly severe depression, and a score of 20–27 indicated severe depression, respectively. Also, based on DSM-IV criteria, Generalized Anxiety Disorder (GAD-7) was used to measure anxiety disorder [19]. Respondents with scores of 5 to 9 were classified as having mild anxiety, 10 to 15 as having moderate anxiety, and 15 or more as having severe anxiety. To identify PTSD, anxiety, and depression in the respondents, these questionnaires were administered in English and was also translated in the local language Malayalam for better understanding. The trained enumerators, who were fluent in Malayalam and English, asked the survey questions face-to-face whilst strict privacy was maintained.

### Variables

The study variables included sociodemographic factors such age, gender, marital status, family structure, level of education, employment status, chronic illness, monthly income, and location. The flood exposure factors were water levels at homes during flooding, injury during flooding, loss of family member, loss of land or flood damage to property, and social support or financial help post event. The primary outcome variable was psychiatric morbidity (PTSD, anxiety and depression).

### Ethical considerations

All study participants were explained about the study and their informed consent was obtained in English or Malayalam. Anonymous data were recorded on the questionnaire. Ethical approval was obtained from the Research Ethics Committee in the School of Behavioral Sciences, Mahatma Gandhi University, India.

### Statistical analysis

Data were presented as proportions, medians (range), or mean (± standard deviation; SD) as appropriate. The variables of interest such as socio-demographic variables and the intensity of flood exposure were compared and analyzed according to the PTSD diagnosis, gender, PCL-5 subscale in patients with subclinical PTSD, level of anxiety and depression. Differences in categorical and continuous variables were analyzed using the χ2 test and students t-test, as appropriate. The Pearson correlation coefficient (r) was calculated to identify the linear relationship between the psychometric parameters. A significant difference was considered when the 2-tailed p-value was less than 0.05. A one-way multivariate analysis of covariance (MANCOVA) was performed to determin the effect of gender on the adjusted means of total score of PTSD, GAD-7 and PHQ-9 with age as covariate. Data were analyzed using the Statistical Package for Social Sciences version 21 (SPSS Inc., Chicago, IL).

## Results

A total of 276 respondents (150 males and 126 females) agreed to participate, their mean age was 46.0±15.2 years and the majority were married (74%), belonged to nuclear families (93.5%) and lived in urban areas (83.3%). **Table 1** displays the sociodemographic details of the study subjects. Seventy percent of respondents had reached primary/middle/high school education, while 24.6% were graduates or diploma holders. Nearly two-thirds (65.5%) of respondents had an average monthly household income ≤4,000 Indian Rupees (IR). Most had >2 feet water level at the home (82.4%) during the flood. Nearly half (n=120/ 43.5%) did not receive any financial help after the flood. Subclinical PTSD symptoms were identified in the majority of participants. Those with subclinical PTSD were six years older in age (46.7±15.3 vs. 40.3±12.2; p=0.03) and were more likely to be hailing from a rural area (97.8% vs. 2.2%; p=0.04). The two groups were comparable for gender, marital status, educational status and employment.

**Table 2** shows the mean PCL-5 with subscale scores i.e. intrusion, arousal and negative cognition, hyperarousal subscales. The mean total score on PCL-5 was 7.89±5.37, and the mean scores for the symptom clusters was highest for intrusion 4.22±3.45, followed by arousal and reactivity (3.10±1.76), negative cognition and mood (2.67±1.46) and avoidance score (1.67±0.93). Those who developed subclinical PTSD had significantly higher levels of GAD-7 (p=0.001) and were more likely to develop mild and moderate anxiety compared to those who did not develop PTSD. Similarly, the score for depression was significantly higher among the subclinical PTSD group [2 (0-12) vs. 0 (0-4), p=0.001] than the non-PTSD group. The frequency of mild and moderate depression was higher in the PTSD group though the difference was not statistically significant.

**Table 3** showed the comparison of gender with respect to the PTSD checklist for DSM-V, GAD-7 and PHQ-9 scores. Females had significantly higher total PTSD symptom severity score (8.24±5.88 vs. 6.07±5.22; p=0.001), higher severity of symptoms of intrusion (4.66±3.60 vs. 3.69±3.20; p=0.04), increased level of anxiety (2.54±1.94 vs. 1.79±1.53; p=0.001) and depression (3.02±2.26 vs. 2.04±1.67; p=0.001) as compared to males. Avoidance symptoms, negative thoughts, and mood, as well as arousal and reactivity, did not differ by gender.

**Table 4** compares the socio-demographic characteristics based on symptom clusters in patients with subclinical PTSD. Elderly individuals (≥60 years) were more likely to have persistent intrusive memories, negative cognition, arousal and avoidance of stimuli than younger participants. Negative cognition, and avoidance of stimuli were more common among unmarried participants, intrusive memories were more frequent in divorced participants and arousal and reactivity was most prevalent among married individuals.

All symptom clusters were more prevalent among the those who were living alone, illiterate, unemployed and those with a prior history of chronic illness. Those with persistent symptoms of avoidance of stimuli were more likely to develop higher level of anxiety (3.06±2.02) and depression (3.37±1.99).

**Table 5** compares levels of anxiety for different socio-demographic characteristics. Moderate level of anxiety was significantly associated with advanced age (68.7±12.5 years; p=0.001) and being illiterate. Whereas, individuals who were unemployed and lived alone were more likely to develop minimal level of anxiety. Moreover, there was a significant relationship between moderate level of anxiety with PTSD (20.67±2.08; p=0.001) and depression (7.33±5.50; p=0.001).

**Table 6** compares the socio-demographic characteristics based on severity of depression. Moderate levels of depression were significantly associated with advanced age (58.6±16.3 years; p=0.005), higher PTSD score (13.0±7.51; p=0.006) and level of anxiety (5.40±4.72; p=0.001). On the other hand, mild level of depression was more likely to be observed in those who were unmarried or divorced, living alone, or were unemployed.

**Table 7** shows bivariate correlation between psychometric parameters. A significant correlation was observed between total score on PCL-5 and GAD-7 (r=0.339, p=0.001) and PHQ-9 (r=0.262, p=0.001).

The one-way MANCOVA showed no statistically significant difference between the gender on the adjusted means of total scores of PTSD, GAD-7 and PHQ-9 after controlling for age.

## Discussion

This study attempted to find the psychological impact of flood and possible factors associated with it in adults in Kerala 2018 flood victims. This is one of the few studies exploring long-term sequalae of flood on the psychological well-being, including PTSD, anxiety and depression.

### Long-term sequalae of mental health illness

We found that flooding has a negative influence on mental health that lasted at least a year after the flooding, accompanied with a higher prevalence of psychological morbidity, notably anxiety, depression, and PTSD, which is in agreement with findings of earlier studies [20, 21].

In our study, female flood survivors had higher score of PTSD, anxiety, and depression than males as reported elsewhere [22-24]. Moreover, we also observed symptom clusters in patients with subclinical PTSD which are significantly associated with depression and anxiety. Flood exposure may trigger PTSD and the victims are likely to experience symptoms related to intrusion, avoidance, negative cognition and mood, and arousal and reactivity. Additionally, these emotions may result in a vicious cycle of thinking among the victims, which in turn could lead to higher levels of depression and anxiety. Together, our findings suggest that PTSD symptoms, rather than depression and anxiety, are more closely associated with flood exposure [12]. Following disasters, it is also common to record cases of depression, anxiety disorders, and substance misuse problems, which are frequent co-morbidities of PTSD [25]. Exposure to flooding has been reported as linked to PTSD, anxiety, and depressive symptoms [26,27]. It has been reported that flood victims exhibit profound depressive symptoms, anxiety, stress, and emotional distress than those who were not exposed to flood [28,29]. Our study also found an association between selected PTSD symptoms and various demographic factors, such as age, gender, marital status and education levels. We found that females had significantly higher total PTSD symptom severity scores, higher severity of symptoms of intrusion, increased level of anxiety and depression than males. However, the gender difference for PTSD symptoms disappeared when controlling for age. Consistent with our findings, a previous study showed a high mean score for all subscales of PTSD i.e. intrusion, avoidance, and hyperarousal among females than those in the males [30].

According to a previous meta-analysis, PTSD was strongly predicted by the severity of the trauma, a lack of social support, and additional life stressors [31]. Therefore, the use of more precise and impartial measures of psychiatric illness would help to mitigate the long-term impact of flooding. The severity of the flood and the length of the follow-up period could have had a significant impact on the variations in prevalence [32]. After a traumatic event, it is well known that the prevalence of PTSD and anxiety declines over time [33].

A relationship between depression, anxiety and PTSD among those who have experienced natural disasters has been reported with higher probable depression (aOR: 7.77), anxiety (aOR: 4.16), and PTSD (aOR: 14.41) [34]. The present study demonstrated a significant correlation between total score on PTSD with anxiety (r=0.339) and depression (r=0.262).

### Psychosocial post disaster care

Those affected by flood or other disasters are vulnerable to a sudden stress reaction, which can result in the development of PTSD. An earlier study on PTSD in flood survivors of Kerala flood 2018 highlighted the need to carry out post-disaster mental health screening because of the high prevalence of PTSD following the floods [16]. Similarly, another study revealed that even eight months post disaster, a sizable percentage of adolescents were still experiencing stress-related disorders [15]. The health department of Kerala trained close to 1000 volunteers to help affected flood victims. Many qualified psychiatrists from various hospitals went to camps to provide flood survivors with psychosocial support, whilst helplines were also opened. As Kerala is the only Indian state with a District Mental Health Program (DMHP) that is accessible in every district, this was made possible [35]. Utilizing 122 intervention teams, the Mental Health Disaster Management team visited 1.23 lakh homes and 706 camps in flood-affected districts. Psychosocial intervention was given to 2.04 lakh individuals and 1543 individuals received pharmacotherapy [36]. Accredited social health activist (ASHA) workers were mobilized, trained, and then sent to multiple relief camps to assist flood victims. Since small health units' emergency plans heavily rely on the concept of hospital networking, these units expanded and used their existing capabilities to assist damaged hospitals. In view of this, there is a need to transform healthcare facility infrastructure and revamp its operational preparedness to mitigate climate change threats following stressful climate events [37]. Identifying issues and gaps in climate preparedness for efficient health delivery, including pre-planning and post disaster response is the key to climate change risk mitigation.

### Strengths and limitations of this study

Our study further adds to the existing knowledge and has addressed gaps about the effects of floods on mental health in Kerala, India. Utilizing standardized tools, the three main mental health outcomes i.e. depression, anxiety, and PTSD were evaluated. The following limitations need to be taken into account, first as a cross-sectional study, it is impossible to draw inferences about cause-and-effect relationships. Therefore, longitudinal studies that could indicate effects in both directions are more appropriate. Future studies that incorporate data from behavioural assessments may provide further insights. Although, we calculated the sample size, our fairly small sample may have resulted in less precise estimations in our population. Therefore, further research with larger samples would be required to offer more genarabisable findings. Despite these limitations, the current study advances our knowledge of the post-flood related psychological consequences and adds to the body of literature in several ways. The psychological well-being of flood victims might be improved by efforts to train medical practitioners to deliver psychotherapy interventions given the possibility of adverse mental health outcomes, such as symptoms of PTSD and depression following floods.

## Conclusion

Our findings highlighted that the vast majority of respondents experienced symptoms of subclinical psychiatric illness that persisted even one year post-flood. Within this population we found differences for selected PTSD symptoms by gender, age, marital status and education levels. Hence, we conclude that tailored psychological interventions are warranted to counter the long-lasting impact of flooding on the mental health of individuals.

## Figures and Tables

**Table 1:** Socio-demographic characteristics of flood survivors by PTSD symptoms

Variables	Overall (n=276)	No-PTSD (n=29)	Subclinical PTSD (n=247)	P value
**Age (mean±SD, years)**	46.0±15.2	40.3±12.2	46.7±15.3	0.03**
**18-34**	64 (23.2%)	10 (15.6%)	54 (84.4%)	0.08 for all
**35-44**	63 (22.8%)	7 (11.1%)	56 (88.9%)	
**45-59**	93 (33.7%)	11 (11.8%)	82 (88.2%)	
**≥60**	56 (20.3%)	1 (1.8%)	55 (98.2%)	
**Males**	150 (54.3%)	19 (12.7%)	131 (87.3%)	0.20
**Females**	126 (45.7%)	10 (7.9%)	116 (92.1%)	
**Marital status (n=274)**				
**Unmarried**	52 (19.0%)	9 (17.3%)	43 (82.7%)	0.21
**Married**	203 (74.0%)	18 (8.9%)	185 (91.1%)	
**Divorced/widowed**	19 (7.0%)	2 (10.5%)	17 (89.5%)	
**Family Type (n=275)**				
**Nuclear**	257 (93.5%)	28 (10.9%)	229 (89.1%)	0.71
**Joint**	14 (5.1%)	1 (7.1%)	13 (92.9%)	
**Alone**	4 (1.5%)	0 (0.0%)	4 (100%)	
**Educational Status**				
**Graduate/Diploma**	68 (24.6%)	10 (14.7%)	58 (85.3%)	0.29
**Primary-to-high school**	200 (72.5%)	19 (9.5%)	181 (90.5%)	
**Illiterate**	8 (2.9%)	0 (0.0%)	8 (100%)	
**Unemployed/Retired**	105 (38.0%)	8 (7.6%)	97 (92.4%)	0.22
**Employed**	171 (62.0%)	21 (12.3%)	150 (87.7%)	
**Monthly Family Income (IR)**				
**20,001– 30,000**	2 (0.7%)	0 (0.0%)	2 (100%)	0.03**
**12,001 – 20,000**	10 (3.6%)	0 (0.0%)	10 (100%)	
**4,001 – 12,000**	82 (29.8%)	7 (8.8%)	75 (91.5%)	
**≤ 4,000**	180 (65.5%)	21 (11.7%)	159 (88.3%)	
**Poor previous health status**	19 (6.9%)	2 (10.5%)	17 (89.5%)	0.14
**Locality (n=275)**				
**Urban**	229 (83.3%)	28 (12.2%)	201 (87.8%)	0.04**
**Rural**	46 (16.7%)	1 (2.2%)	45 (97.8%)	
**Flood exposure factors**				
**Water level at the home (feet)**	4 (1-6)	4 (2-5)	4 (1-6)	0.03**
**≤2**	48 (17.6%)	1 (2.1%)	47 (97.9%)	0.03**
**>2**	224 (82.4%)	28 (12.5%)	196 (87.5%)	
**Injury due to flooding**	3 (1.1%)	0 (0.0%)	3 (100%)	0.55
**Loss of family member**	2 (0.7%)	0 (0.0%)	2 (100%)	0.62
**No financial help**	120 (43.5%)	8 (6.7%)	112 (93.3%)	0.24

** statistically significant

**Table 2:** Psychometric parameters of PTSD symptoms (subscale scores), Anxiety & Depression scores

Variables	Overall (n=276)	No-PTSD (n=29)	Subclinical PTSD (n=247)	P value
**PCL-5 total score**	7.89±5.37	**-**	7.89±5.37	**-**
**PCL-5 subscales**				
**Intrusion**	4.22±3.45	**-**	4.22±3.45	**-**
**Avoidance**	1.67±0.93	**-**	1.67±0.93	**-**
**Negative cognition and mood**	2.67±1.46	**-**	2.67±1.46	**-**
**Arousal and reactivity**	3.10±1.76	**-**	3.10±1.76	**-**
				
**Generalized anxiety disorder (level of anxiety)**	2 (2-11)	0 (0-2)	2 (0-11)	0.001**
**Minimal (0-4)**	258 (93.5%)	29 (100%)	229 (92.7%)	0.32
**Mild (5-9)**	15 (5.4%)	0 (0.0%)	15 (6.1%)	
**Moderate (10-15)**	3 (1.1%)	0 (0.0%)	3 (1.2%)	
				
**Patient health questionnaire 9**	2 (0-12)	0 (0-4)	2 (0-12)	0.001**
**None-minimal depression (0-4)**	245 (88.8%)	29 (100%)	216 (87.4%)	0.12
**Mild depression (5-9)**	26 (9.4%)	0 (0.0%)	26 (10.5%)	
**Moderate depression (10-14)**	5 (1.8%)	0 (0.0%)	5 (2.0%)	

** statistically significant

**Table 3:** PTSD Checklist for DSM-V, GAD-7 and PHQ-9 scores by gender

Variables	Females (n=126)	Males (n=150)	P value
**Age**	45.86±15.67	46.20±14.64	0.85
**PCL-5 total score**	8.24±5.88	6.07±5.22	0.001**
**PCL-5 subscales**			
**Intrusion**	4.66±3.60	3.69±3.20	0.04**
**Avoidance**	1.70±0.94	1.63±0.93	0.68
**Negative cognition and mood**	2.83±1.43	2.50±1.47	0.14
**Arousal and reactivity**	3.38±2.15	2.94±1.49	0.18
			
**Generalized anxiety disorder (level of anxiety)**	2.54±1.94	1.79±1.53	0.001**
**Minimal (0-4)**	114 (90.5%)	144 (96.0%)	0.18
**Mild (5-9)**	10 (7.9%)	5 (3.3%)	
**Moderate (10-15)**	2 (1.6%)	1 (0.7%)	
			
**Patient health questionnaire- 9**	3.02±2.26	2.04±1.67	0.001**
**None-minimal depression (0-4)**	106 (84.1%)	139 (92.7%)	0.06**
**Mild depression (5-9)**	16 (12.7%)	10 (6.7%)	
**Moderate depression (10-14)**	4 (3.2%)	1 (0.7%)	

** statistically significant

**Table 4:** Symptom clusters in patients with subclinical PTSD (n=247) by demographics

Variables	Intrusion (n=202)	Avoidance (n=108)	Negative cognition and mood (n=162)	Arousal and reactivity (n=156)
**Age (mean±SD, years)**	47.1±16.1	48.7±17.3	47.5±16.5	47.3±15.6
**18-34**	45 (70.3%)	23 (35.9%)	37 (57.8%)	33 (51.6%)
**35-44**	44 (69.8%)	22 (34.9%)	32 (50.8%)	35 (55.6%)
**45-59**	62 (66.7%)	31 (33.3%)	50 (53.8%)	50 (53.8%)
**≥60**	51 (91.1%)	32 (57.1%)	43 (76.8%)	38 (67.9%)
**Females**	111 (88.1%)	57 (45.2%)	84 (66.7%)	55 (43.7%)
**Males**	91 (60.7%)	51 (34.0%)	78 (51.9%)	101 (67.3%)
**Marital status (n=274)**				
**Unmarried**	39 (75.0%)	26 (50.0%)	33 (63.5%)	29 (55.8%)
**Married**	146 (71.9%)	73 (36.0%)	118 (58.1%)	119 (58.6%)
**Divorced/widowed**	15 (78.9%)	8 (42.1%)	10 (52.6%)	8 (42.1%)
**Family Type (n=275)**				
**Nuclear**	186 (72.4%)	102 (39.7%)	150 (58.4%)	145 (56.4%)
**Joint**	11 (78.6%)	2 (14.3%)	7 (50.0%)	7 (50.0%)
**Alone**	4 (100%)	4 (100%)	4 (100%)	4 (100%)
**Educational Status**				
**Graduate/Diploma**	56 (82.4%)	36 (52.9%)	49 (72.1%)	40 (58.8%)
**Primary-to-high school**	139 (69.5%)	67 (33.5%)	107 (53.5%)	109 (54.5%)
**Illiterate**	7 (87.5%)	5 (62.5%)	6 (75.0%)	7 (87.5%)
**Unemployed/Retired**	93 (88.6%)	49 (46.7%)	77 (73.3%)	54 (51.4%)
**Employed**	109 (63.7%)	59 (34.5%)	85 (49.7%)	102 (59.6%)
**Monthly Family Income (IR)**				
**20,001– 30,000**	2 (100%)	2 (100%)	2 (100%)	2 (100%)
**12,001 – 20,000**	10 (100%)	7 (70.0%)	7 (70.0%)	7 (70%)
**4,001 – 12,000**	75 (91.5%)	28 (34.1%)	60 (73.2%)	38 (46.3%)
**≤ 4,000**	114 (63.3%)	70 (38.9%)	92 (51.1%)	108 (60.0%)
**Chronic illness**	15 (78.9%)	10 (52.6%)	11 (57.9%)	12 (63.2%)
**Water level at the home (feet)**				
**≤2**	40 (83.3%)	12 (25.0%)	35 (72.9%)	27 (56.3%)
**>2**	160 (71.4%)	94 (42.0%)	124 (55.4%)	126 (56.3%)
**Injured**	3 (100%)	0 (0.0%)	1 (33.3%)	1 (33.3%)
**No financial help**	99 (65.8%)	39 (32.5%)	84 (70.0%)	58 (48.3%)
**Loss of family member**	2 (100%)	1 (50.0%)	1 (50.0%)	0 (0.0%)
**Generalized anxiety disorder**	2.57±1.77	3.06±2.02	2.74±1.84	2.52±1.97
**Minimal (0-4)**	184 (71.3%)	96 (37.2%)	147 (57.0%)	143 (5.4%)
**Mild anxiety (5-9)**	15 (100%)	9 (60.0%)	12 (80.0%)	10 (66.7%)
**Moderate anxiety (10-15)**	3 (100%)	3 (100%)	3 (100%)	3 (100%)
**Patient health questionnaire- 9**	2.94±1.96	3.37±1.99	3.08±2.07	2.72±2.02
**Minimal depression (1-4)**	172 (70.2%)	86 (35.1%)	134 (54.7%)	135 (55.1%)
**Mild depression (5-9)**	26 (100%)	9 (73.1%)	23 (88.5%)	18 (69.2%)
**Moderate depression (10-14)**	4 (80.0%)	3 (60.0%)	5 (100%)	3 (60.0%)

** statistically significant

**Table 5:** Comparison of socio-demographic characteristics based on level of Anxiety

Variables	Minimal Anxiety (n=258)	Mild Anxiety (n=15)	Moderate Anxiety (n=3)	P value
**Age (mean±SD) years**	45.3±14.9	53.5±13.7	68.7±12.5	0.004**
**18-34**	63 (98.4%)	1 (1.6%)	0 (0.0%)	0.006**
**35-44**	61 (96.8%)	2 (3.2%)	0 (0.0%)	
**45-59**	87 (93.5%)	6 (6.5%)	0 (0.0%)	
**≥60**	47 (83.9%)	6 (10.7%)	3 (5.4%)	
**Males**	144 (96.0%)	5 (3.3%)	1 (0.7%)	0.18
**Females**	114 (90.5%)	10 (7.9%)	2 (1.6%)	
**Marital status (n=274)**				
**Unmarried**	47 (90.4%)	3 (5.8%)	2 (3.8%)	0.06**
**Married**	193 (95.1%)	9 (4.4%)	1 (0.5%)	
**Divorced/widowed**	16 (84.2%)	3 (15.8%)	0 (0.0%)	
**Family Type (n=275)**				
**Nuclear**	244 (94.9%)	11 (4.3%)	2 (0.8%)	0.001**
**Joint**	12 (85.7%)	1 (7.1%)	1 (7.1%)	
**Alone**	2 (50.0%)	2 (50.0%)	0 (0.0%)	
**Educational Status**				
**Graduate/Diploma**	66 (97.1%)	2 (2.9%)	0 (0.0%)	0.001**
**Primary/middle/high school degree**	187 (93.5%)	12 (6.0%)	1 (0.5%)	
**Illiterate**	5 (62.5%)	1 (12.5%)	2 (25.0%)	
**Unemployed/Retired**	93 (88.6%)	9 (8.6%)	3 (2.9%)	0.01**
**Employed**	165 (96.5%)	6 (3.5%)	0 (0.0%)	
**Monthly Family Income (IR)**				
**20,001– 30,000**	2 (100%)	0 (0.0%)	0 (0.0%)	0.48
**12,001 – 20,000**	8 (80.0%)	2 (20.0%)	0 (0.0%)	
**4,001 – 12,000**	80 (97.6%)	2 (2.4%)	0 (0.0%)	
**≤ 4,000**	166 (92.2%)	11(6.1%)	3 (1.7%)	
**Chronic illness**	15 (78.9%)	3 (15.8%)	1 (5.3%)	0.06
**Water level at the home (in feet)**				
**≤2**	48 (100%)	0 (0.0%)	0 (0.0%)	0. 21
**>2**	206 (92.0%)	15 (6.7%)	3 (1.3%)	
**No financial help**	112 (93.3%)	7 (5.8%)	1 (0.8%)	0.99
**PCL-5 total score**	6.76±5.48	9.47±4.79	20.67±2.08	0.001**
**PCL-5 subscales**				
**Intrusion (Q1-Q5)**	4.14±3.47	4.53±33.18	8.0±1.0	0.14
**Avoidance (Q6-Q7)**	1.66±0.93	1.78±1.09	1.67±0.57	0.93
**Cognition and mood (Q8-Q14)**	2.591.37	2.92±1.44	6.0±2.0	0.001**
**Arousal and reactivity (Q15-Q20)**	3.11±1.70	2.30±1.49	5.0±4.0	0.06
**Patient health questionnaire**	2.31±1.84	4.67±1.67	7.33±5.50	0.001**
**Minimal depression (1-4)**	236 (96.3%)	8 (3.3%)	1 (0.4%)	0.001**
**Mild depression (5-9)**	19 (73.1%)	7 (26.9%)	0 (0.0%)	
**Moderate depression (10-14)**	3 (60.0%)	0 (0.0%)	2 (40.0%)	

** statistically significant

**Table 6:** Comparison of socio-demographic characteristics based on severity of depression

Variables	Minimal depression (n=245)	Mild depression (n=26)	Moderate depression (n=5)	P value
**Age (mean±SD, years**	45.0±14.5	53.3±17.4	58.6±16.3	0.005**
**18-34**	59 (92.2%)	5 (7.8%)	0 (0.0%)	0.001**
**35-44**	61 (96.8%)	1 (1.6%)	1 (1.6%)	
**45-59**	85 (91.4%)	7 (7.5%)	1 (1.1%)	
**≥60**	40 (71.4%)	13 (23.2%)	3 (5.4%)	
**Males**	139 (92.7%)	10 (6.7%)	1 (0.7%)	0.06
**Females**	106 (84.1%)	16 (12.7%)	4 (3.2%)	
**Marital status (n=274)**				
**Unmarried**	39 (75.0%)	11 (21.2%)	2 (3.8%)	0.001**
**Married**	192 (94.6%)	9 (4.4%)	2 (1.0%)	
**Divorced/widowed**	12 (63.2%)	6 (31.6%)	1 (5.3%)	
**Family Type (n=275)**				
**Nuclear**	229 (89.1%)	24 (9.3%)	4 (1.6%)	0.02**
**Joint**	13 (92.9%)	0 (0.0%)	1 (7.1%)	
**Alone**	2 (50.0%)	2 (50.0%)	0 (0.0%)	
**Educational Status**				
**Graduate/Diploma**	63 (92.6%)	5 (7.4%)	0 (0.0%)	1.00
**Primary- to-high school**	175 (87.5%)	21 (10.5%)	4 (2.0%)	
**Illiterate**	7 (87.5%)	0 (0.0%)	1 (12.5%)	
**Unemployed/Retired**	85 (81.0%)	15 (14.3%)	5 (4.8%)	0.001**
**Employed**	160 (93.6%)	11 (6.4%)	0 (0.0%)	
**Monthly Family Income (IR)**				
**20,001– 30,000**	2 (100%)	0 (0.0%)	0 (0.0%)	0.46
**12,001 – 20,000**	10 (100%)	0 (0.0%)	0 (0.0%)	
**4,001 – 12,000**	78 (95.1%)	3 (3.7%)	1 (1.2%)	
**≤ 4,000**	153 (85.0%)	23 (12.8%)	4 (2.2%)	
**Chronic illness**	15 (78.9%)	2 (10.5%)	2 (10.5%)	0.02**
**Water level at the home (feet)**				
**≤2**	43 (89.6%)	4 (8.3%)	1 (2.1%)	0.94
**>2**	198 (88.4%)	22 (9.8%)	4 (1.8%)	
**No financial help**	112 (93.3%)	6 (5.0%)	2 (1.7%)	0.19
**Generalized anxiety disorder**	1.89±1.51	3.96±2.12	5.60±4.98	0.001**
**Minimal (0-4)**	236 (91.5%)	19 (7.4%)	3 (1.2%)	0.001**
**Mild anxiety (5-9)**	8 (53.3%)	7 (46.7%)	0 (0.0%)	
**Moderate anxiety (10-15)**	1 (33.3%)	0 (0.0%)	2 (66.7%)	
**PCL-5 total score**	6.72±5.54	9.15±5.18	13.0±7.51	0.006**
**PCL-5 subscales**				
**Intrusion (Q1-Q5)**	4.29±3.58	3.23±1.86	7.75±3.40	0.04**
**Avoidance (Q6-Q7)**	1.64±0.95	1.79±0.91	1.67±0.57	0.82
**Cognition and mood (Q8-Q14)**	2.64±1.42	2.48±1.31	4.40±2.19	0.02**
**Arousal and reactivity (Q15-Q20)**	3.06±1.75	3.50±1.75	2.33±2.30	0.46

** statistically significant

**Table 7:** Bivariate correlation between psychometric parameters

Variables		PTSD total score	GAD-7 total score	PHQ-9 total score
**PTSD total score**	**Pearson Correlation**	1	0.339^**^	0.262^**^
	**Sig. (2-tailed)**		0.001	0.001
**GAD-7 total score**	**Pearson Correlation**	0.339^**^	1	.568^**^
	**Sig. (2-tailed)**	0.001		0.001
**PHQ-9 total score**	**Pearson Correlation**	0.262^**^	0.568^**^	1
	**Sig. (2-tailed)**	0.001	0.001	

** statistically significant
